# Colonic involvement in disseminated histoplasmosis of an immunocompetent adult: case report and literature review

**DOI:** 10.1186/1471-2334-13-143

**Published:** 2013-03-20

**Authors:** Biwei Yang, Lixia Lu, Dajiang Li, Li Liu, Libin Huang, Liyu Chen, Hong Tang, Lichun Wang

**Affiliations:** 1 Center of Infectious Diseases, Division of Molecular Biology of Infectious Diseases, State Key Laboratory of Biotherapy (Sichuan University), West China Hospital, Sichuan University, NO. 37, Guoxue lane, Wuhou District, Chengdu 610041, China; 2 Department of Gastroenterology, West China Hospital, Sichuan University, NO. 37, Guoxue lane, Wuhou District, Chengdu 610041, China

**Keywords:** Disseminated histoplasmosis, Immunocompetence, Colonic involvement

## Abstract

**Background:**

Histoplasmosis is a common opportunistic fungal infection that is observed almost exclusively in immunodeficient patients, especially those with AIDS. Immunocompetent individuals that suffer from histoplasmosis are rarely reported, especially those with disseminated lesions, such as disseminated histoplasmosis. The observation of disseminated histoplasmosis with prominent gastrointestinal involvement, no respiratory symptoms (which is presumed to be the portal of infection), gastrointestinal pathological changes, and minor digestive system disorders make this case study exceedingly rare.

**Case presentation:**

We report the case of a 33-year-old immunocompetent male who presented with fever and weight loss. Based on investigations, the patient showed pancytopenia, hepatosplenomegaly, bone marrow involvement and marked colonic involvement. Finally, disseminated histoplasmosis was diagnosed and confirmed by stained smears of fine needle aspirates and biopsy from lesions in the bone marrow and colon. The patient showed appreciable regression of lesions following prompt treatment with amphotericin B deoxycholate, and was treated thereafter with oral itraconazole following discharge from hospital.

**Conclusion:**

Disseminated histoplasmosis could be underestimated in immunocompetent patients. A high degree of clinical suspicion is essential in both immunocompromised and immunocompetent patients, regardless of pulmonary symptoms, and whether in endemic or non-endemic areas. Early and accurate diagnosis is extremely important for the appropriate treatment of infection and to improve disease outcome.

## Background

Histoplasmosis, an invasive fungal infection that commonly occurs in immunodeficient patients, mainly manifests as pulmonary lesions or as disseminated patterns with multiple organ involvement in some severe cases. Interestingly, cases of histoplasmosis in immunocompetent hosts have rarely been reported, especially those accompanied with disseminated histoplasmosis and marked colonic involvement. The present report illustrates the case of a young male patient without any identifiable immunodeficiency diseases who presented with disseminated histoplasmosis with prominent manifestations of colonic involvement.

## Case presentation

A 33-year-old male patient was admitted to West China Hospital in March 2012 with a history of intermittent moderate fever with associated night sweats, anorexia for more than 3 months’ duration and weight loss of 15 kg. His fever occurred once or twice daily with no chills and lasted about 2 h and then returned to normal levels automatically with recorded temperatures between 36.5°C and 38.5°C. There was no obvious cough or expectoration. His past medical history suggested no underlying diseases and he denied any travel to the endemic area of Kala-azar or history of histoplasmosis but mentioned an occasional damp working environment.

On admission, the patient’s heart rate was 94/min, temperature was 37.7°C, respiration 25/min, and blood pressure 96/64 mmHg. The spleen was 5 cm below the left rib border and the liver was impalpable by physical examination. Laboratory investigations upon admission revealed slight pancytopenia (hemoglobin 9.8 g/dL, hematocrit 0.30 L/L, platelet count 92,000/mm^3^ and white blood cell count 3,200/mm^3^), elevated erythrocyte sedimentation rate (ESR) of 61 mm at the end of 1 h and increased alkaline phosphatase to 277 IU/L. Lymphocyte subsets comprised 47.13% CD4, 28.42% CD8, CD4/CD8 ratio of 1.66 and CD4 counts of 240/mm^3^. Immunoglobulin levels comprised IgG 22.80 g/L (normal range 8–15.5), IgA 2860.00 mg/L (range 836–2900), IgM 564.00 mg/L (range 700–2200), normal complement levels with C3 0.88 g/L (normal range 0.785-1.52), C4 0.2360 g/L (normal range 0.145-0.36) and increased CIC 0.33 optical density (OD) (normal <0.15). Liver enzymes and kidney function tests and other blood tests including inflammatory markers (PCT, IL-6, CRP, SAA), tumor markers (AFP, CEA, CA19-9, CA-125), autoantibodies, antibodies related to infectious diseases including hepatitis B virus, hepatitis C virus, and repeatedly performed human immunodeficiency virus (HIV) tests during the hospital stay were normal or negative. Routine urine tests were normal but fecal occult blood test (FOBT) for hidden blood in the stool was weakly positive.

Chest computed tomography (CT) scans were normal. However, abdominal CT scan and ultrasonography both revealed hepatosplenomegaly. However, repeated blood, sputum, urine, stool and bone marrow cultures were negative.

**Figure 1:**
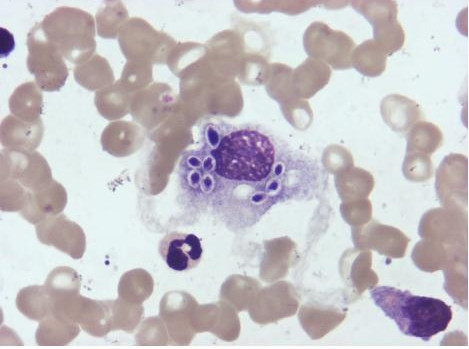
Hematoxylin and eosin stained bone marrow aspirate.

Hematoxylin and eosin stained bone marrow aspirate demonstrated oval or round organisms with amaranth nuclei and capsule-like unstained halos around them, confined to the cytoplasm of phagocytes, which is highly suggestive of *Histoplasmosis capsulatum* (Figure 1). Sections of bone marrow biopsy stained with PAS (periodic acid-schiff) and GMS (Gomori methenamine silver) were both positive, which was compatible with a diagnosis of histoplasmosis.

**Figure 2:**
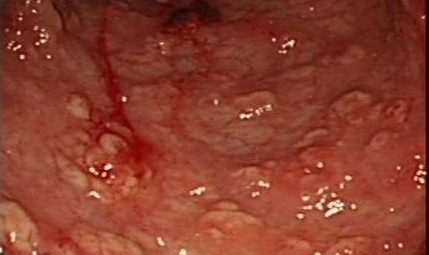
Transverse colon seen during endoscopy.

**Figure 3:**
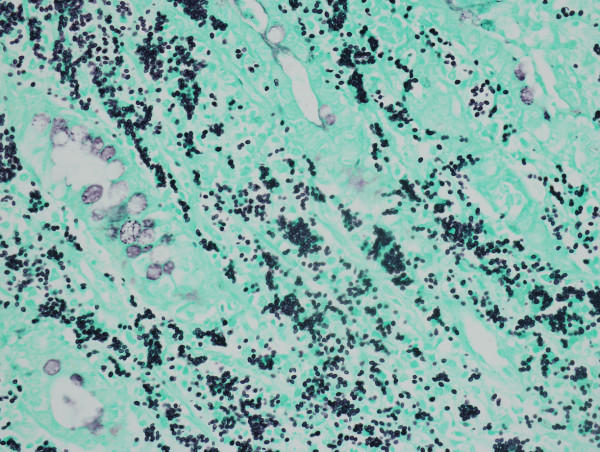
Gomori methenamine silver (GMS) stained colon biopsy specimen (magnification × 40).

Gastrointestinal endoscopy was subsequently performed, and colonoscopy showed strictures, edematous mucosa and diffuse aphthoid ulcers measuring 0.5-1.0 cm in diameter scattered throughout the mucous membrane of the colon (Figure 2). Biopsy specimens of the colon identified numerous yeast-like structures within increased numbers of histiocytes staining positive for PAS stain, and GMS stain but Giemsa stain negative (Figure 3), indicating granulomatous inflammation induced by mycotic infection, which was consistent with histoplasmosis.

The patient was started on intravenous amphotericin B deoxycholate at an initial dose of 1 mg/d increasing to 5 mg a day until a dose of 50 mg (approximately 0.8 mg/kg) once daily was reached. One week after treatment, the patient’s temperature returned to normal levels, and his appetite improved. More than 1 month later, the follow-up CT scan showed the spleen had returned to a normal size, and *H. capsulatum* was not observed in bone marrow aspirate. Enteroscopy performed 2 months after treatment showed that the ulcers and congestion of mucosa were recovering. A blood test before the patient was discharged showed hemoglobin levels of 12.3 g/dL, platelet counts of 179,000/mm^3^ and WBCs 6,150/mm^3^ with an absolute lymphocyte count (ALC) of 1.51 × 10^9^/L and CD4 count of 493/mm^3^.

Amphotericin B deoxycholate treatment was administered for 2 months with a total quantity used of 2000 mg. The treatment was then shifted to oral itraconazole (200 mg, twice daily) before the patient was discharged.

## Discussion

Histoplasmosis, caused by *H. capsulatum*, is generally seen in immunocompromised hosts, especially HIV/AIDS patients [1,2]. It also occurs in immunocompetent individuals and frequently presents as an asymptomatic or self-limited influenza-like process. In several reported cases, serious extrapulmonary injury occurred in groups of children [3,4], or patients with underlying diseases [5,6]. Infection occurs by inhalation of fungal spores and results in a variety of clinical manifestations including acute pulmonary histoplasmosis, chronic pulmonary histoplasmosis or disseminated histoplasmosis [7]. In some instances it undergoes a latent period, such as in the case of lung reactivation of a latent histoplasmosis that occurred more than 45 years after exposure [8]. The patient in the current case study is a healthy young man without any identifiable immunodeficiency. We regard the transient low CD4 counts as an outcome rather than cause of infection, as the counts increased when the systematic infection was treated. He presented with disseminated histoplasmosis, yet lacked radiographic evidence of pulmonary involvement.

Histoplasmosis can infect multiple systems, including the central nervous system [9], articulation [6], and bone marrow, as well as nasal septum [10] and skin [11]. Occasionally it can lead to variable symptoms or even fatal outcomes, such as adrenal insufficiency induced by adrenal gland lesions [12], dysphagia induced by laryngopharyngeal nodular mass [5,13] or renal failure induced by granulomatous interstitial nephritis [14]. All the symptoms described above can be observed as isolated focal lesions or as one aspect of systemic dissemination. This case showed systemic dissemination involving bone marrow, spleen and colon, of which the intestinal pathologic changes were greatest.

Gastrointestinal (GI) involvement occurs in 70 to 90% of cases with disseminated histoplasmosis [15], involving the stomach, duodenum and colon [16]. It can appear with a variety of clinical presentations, from ulceration and GI bleeding to bowel obstruction and bowel perforation in AIDS patients [17] but generally is asymptomatic or presents with vague abdominal symptoms [18]. This case demonstrated gastrointestinal involvement that only occurred in the colon without gastrointestinal symptoms such as vomiting, diarrhea and constipation but with a weakly positive FOBT. Pathologic changes of the colon were prominent and severe at first, but the scattered ulcers, congestion and edema of mucosa all recovered after amphotericin B deoxycholate therapy.

Currently, treatment with amphotericin B deoxycholate is only recommended for severe pulmonary or disseminated disease and then only as an initial therapy, with a shift to an azole agent when the patient’s symptoms improve [19]. Itraconazole is the preferred choice for treatment of mild to moderate histoplasmosis [19]. Voriconazole, for the treatment of relapse after initial therapy with itraconazole [4] is another treatment option. In the current study, we used amphotericin B deoxycholate to treat the patient. The follow-up analysis demonstrated weight gain, body temperature returned to normal, slightly shrunken spleen, unremarkable bone marrow aspirate with no detectable pathogens present, and improved colonic lesions, indicating a good response to the treatment. Treatment was shifted to oral itraconazole for at least half a year, as the total amount of amphotericin B deoxycholate had reached a maximum of 2000 mg.

In summary, this case is notable as the patient is a healthy young man without any immunodeficiency who presented with disseminated histoplasmosis involving bone marrow, spleen and colon, yet lacking evidence of pulmonary involvement. Another remarkable feature of this case is that the marked colonic involvement does not correlate with the absence of gastrointestinal symptoms. Unfortunately, the bone marrow culture was negative, possibly because of limited laboratory conditions and failing to use the required special culture medium.

## Conclusions

Given the appropriate clinical context, disseminated histoplasmosis should be considered in both immunocompromised and immunocompetent patients, regardless of pulmonary symptoms, in endemic or non-endemic areas [20]. Moreover, gastroenterological endoscopy helps to further determine diagnosis and to evaluate prognosis even though no significant gastrointestinal symptoms are observed in disseminated histoplasmosis. Although *H. capsulatum* can be observed with distinctive capsule-like structures during microscopic examination, the specific staining of key tissues is particularly important for determining the species of pathogen when culture information is unobtainable.

### Informed consent

Written informed consent was obtained from the patient for publication of this case report and any accompanying images. A copy of the written consent is available for review by the Editor-in-Chief of this journal.

## Competing interests

The authors declare that they have no competing interests.

## Authors’ contributions

YBW, LLX, LDJ, LL, CLY, TH, and WLC collected the patient data and participated in the treatment. YBW wrote the manuscript. TH and WLC revised and edited the manuscript. HLB performed the gastrointestinal endoscopy. All authors read and approved the final version of the manuscript.

## Pre-publication history

The pre-publication history for this paper can be accessed here:

http://www.biomedcentral.com/1471-2334/13/143/prepub

## References

[B1] Escher M Kainikkara TM Grabner A Ott G Stange EF Herrlinger KR Histoplasmosis: uncommon opportunistic infection in a patient with HIV infection Dtsch Med Wochenschr 2012 137 6 260 264 Epub 2012 Jan 31. German 22294110 10.1055/s-0031-1298872

[B2] Sierra JE Torres JM New clinical and histological patterns of acute disseminated histoplasmosis in human immunodeficiency virus-positive patients with acquired immunodeficiency syndrome Am J Dermatopathol 2011 Epub ahead of print 10.1097/DAD.0b013e31822fd00a 22157244

[B3] Threlkeld ZD Broughton RA Khan GQ Berger JR Isolated histoplasma capsulatum meningoencephalitis in an immunocompetent child J Child Neurol 2012 27 4 532 535 Epub 2012 Jan 12 10.1177/0883073811428780 22241715

[B4] Dhawan J Verma P Sharma A Ramam M Kabra SK Gupta S Disseminated cutaneous histoplasmosis in an immunocompetent child, relapsed with itraconazole, successfully treated with voriconazole Pediatr Dermatol 2010 27 5 549 551 Epub 2010 Aug 26 10.1111/j.1525-1470.2010.01273.x 20796238

[B5] Siraj F Manucha V Pharyngeal histoplasmosis presenting as a tumor mass in an immunocompetent host J Glob Infect Dis 2010 2 1 70 71 10.4103/0974-777X.59256 20300423 PMC2840970

[B6] Makol A Wieland CN Ytterberg SR Articular involvement in disseminated histoplasmosis in a kidney transplant patient taking azathioprine J Rheumatol 2011 38 12 2692 2693 10.3899/jrheum.110776 22134800

[B7] Kauffman CA Histoplasmosis Clin Chest Med 2009 30 2 217 225 v 10.1016/j.ccm.2009.02.002 19375629

[B8] Torres-Rodríguez JM Segura-Roca G Coll J Histoplasmosis 45 years after infection in an immunocompetent man Rev Iberoam Micol 2009 26 4 244 246 Spanish 10.1016/j.riam.2009.03.004 19818663

[B9] Tai YF Kullmann DM Howard RS Scott GM Hirsch NP Revesz T Central nervous system histoplasmosis in an immunocompetent patient J Neurol 2010 257 11 1931 1933 Epub 2010 Jun 22 10.1007/s00415-010-5629-x 20567842

[B10] Oikawa F Carvalho D Matsuda NM Yamada AT Histoplasmosis in the nasal septum without pulmonary involvement in a patient with acquired immunodeficiency syndrome: case report and literature review Sao Paulo Med J 2010 128 4 236 238 21120437 10.1590/S1516-31802010000400012 PMC10938995

[B11] Wobser R Wilpert J Kayser G Walz G Stubanus M Disseminated histoplasmosis with involvement of mediastinum and skin in animmunocompetent patient Dtsch Med Wochenschr 2009 134 12 589 593 Epub 2009 Mar 10. German 10.1055/s-0029-1208090 19277935

[B12] Rana C Krishnani N Kumari N Bilateral adrenal histoplasmosis in immunocompetent patients Diagn Cytopathol 2011 39 4 294 296 10.1002/dc.21416 20607678

[B13] Jana M Hari S Arava SK Disseminated histoplasmosis manifested by laryngopharyngeal and adrenal lesions in an HIV-negative individual J Indian Med Assoc 2010 108 9 615 21510541

[B14] Qian Q Humayun H Humayun Y Sethi S Granulomatous interstitial nephritis associated with disseminated histoplasmosisin an immunocompetent patient Am J Kidney Dis 2011 58 6 1018 1021 Epub 2011 Oct 5 10.1053/j.ajkd.2011.08.022 21974966

[B15] Spivak H Schlasinger MH Tabanda-Lichauco R Ferstenberg H Small bowel obstruction from gastrointestinal histoplasmosis in acquired immune deficiency syndrome Am Surg 1996 62 5 369 372 8615564

[B16] Colaiacovo R de Castro AC Shiang C Ganc RL Ferrari AP Jr Disseminated histoplasmosis: a rare cause of multiple ulcers in the gastrointestinal tract Endoscopy 2011 43 Suppl 2 UCTN E216 Epub 2011 May 16 21590612 10.1055/s-0030-1256398

[B17] Assi M McKinsey DS Driks MR O’Connor MC Bonacini M Graham B Gastrointestinal histoplasmosis in the acquired immunodeficiency syndrome: report of 18 cases and literature review Diagn Microbiol Infect Dis 2006 55 3 195 201 Epub 2006 Mar 20 10.1016/j.diagmicrobio.2006.01.015 16545932

[B18] Goodwin RA Jr Shapiro JL Thurman GH Thurman SS Des Prez RM Disseminated histoplasmosis: clinical and pathological correlations Medicine (Baltimore) 1980 59 1 1 33 7356773

[B19] Wheat LJ Sarosi G McKinsey D Hamill R Bradsher R Johnson P Practice guidelines for the management of patients with histoplasmosis Clin Infect Dis 2000 30 4 688 695 Epub 2000 Apr 20 10.1086/313752 10770731

[B20] Lamps LW Molina CP West AB Haggitt RC Scott MA The pathologic spectrum of gastrointestinal and hepatic histoplasmosis Am J Clin Pathol 2000 113 1 64 72 10.1309/X0Y2-P3GY-TWE8-DM02 10631859

